# A System for Identifying Named Entities in Biomedical Text: how Results From two Evaluations Reflect on Both the System and the Evaluations

**DOI:** 10.1002/cfg.457

**Published:** 2005

**Authors:** Shipra Dingare, Malvina Nissim, Jenny Finkel, Christopher Manning, Claire Grover

**Affiliations:** 1 Institute for Communicating and Collaborative Systems University of Edinburgh 2 Buccleuch Place Edinburgh EH8 9LW UK; 2 Department of Computer Science Stanford University Gates Building 1A 353 Serra Mall Stanford CA 94305-9010 USA

## Abstract

We present a maximum entropy-based system for identifying named entities (NEs) in biomedical abstracts and present its performance in the only two biomedical named entity recognition (NER) comparative evaluations that have been held to date, namely BioCreative and Coling BioNLP. Our system obtained an exact match F-score of 83.2% in the BioCreative evaluation and 70.1% in the BioNLP evaluation. We discuss our system in detail, including its rich use of local features, attention to correct boundary identification, innovative use of external knowledge resources, including parsing and web searches, and rapid adaptation to new NE sets. We also discuss in depth problems with data annotation in the evaluations which caused the final performance to be lower than optimal.


**Comparative and Functional Genomics**
*Comp Funct Genom* 2005; **6**: 77–85.
Published online in Wiley InterScience (www.interscience.wiley.com). **DOI:** 10.1002/cfg.457

**Conference Paper**

### A system for identifying named entities in biomedical text: how results from two evaluations reflect on both the system and the evaluations

Shipra Dingare[1], Malvina Nissim[1] Jenny Finkel[2], Christopher Manning[2]* and Claire Grover[1]
[1] *Institute for Communicating and Collaborative Systems, University of Edinburgh 2 Buccleuch Place,, Edinburgh EH8 9LW, UK*
[2] *Department of Computer Science, Stanford University, Gates Building 1A, 353 Serra Mall, Stanford CA 94305-9010, USA*

**Correspondence to: Christopher Manning, Department of Computer Science, Stanford University, Gates Building 1A, 353 Serra Mall, Stanford CA 94305-9010, USA. E-mail: manning@cs.stanford.edu*

Received: 17 December 2004
Accepted: 22 December 2004

### Abstract

**We present a maximum entropy-based system for identifying named entities (NEs) in biomedical abstracts and present its performance in the only two biomedical named entity recognition (NER) comparative evaluations that have been held to date, namely BioCreative and Coling BioNLP. Our system obtained an exact match F-score of 83.2% in the BioCreative evaluation and 70.1% in the BioNLP evaluation. We discuss our system in detail, including its rich use of local features, attention to correct boundary identification, innovative use of external knowledge resources, including parsing and web searches, and rapid adaptation to new NE sets. We also discuss in depth problems with data annotation in the evaluations which caused the final performance to be lower than optimal.** **Copyright © 2005 John Wiley & Sons, Ltd.**

**Keywords:** **automatic named entity recognition; manual annotation of biomedical texts**

### Introduction

The explosion of information in the biomedical domain has led to immense interest in automated information extraction techniques and consequently to a number of publications describing systems and results for natural language processing tasks on biomedical data. With each group addressing varying tasks, using varying evaluation corpora, and employing varying scoring methods, it has been impossible to properly compare systems and assess the state of progress in the field. The use of standardized evaluations to remedy this state of affairs is only beginning; the Text Retrieval Conference only recently initiated a genomics track to assess biomedical information retrieval and question-answering. Here we focus on the task of Named Entity Recognition (NER), which requires identification of names in shallow semantic categories, such as protein names or drug names. A number of groups have reported results on biomedical NER, attempting to identify anywhere between four and 24 categories, evaluating on corpora ranging from 30 to 100 abstracts and reporting scores varying from 3% for the class 'RNA' to 92% for the specific protein 'SH3' (Collier *et al*., 2000; Fukuda, 1998; Kazama *et al*., 2002; Nobata *et al*., 1999). Recently, two comparative evaluations have been held to evaluate the state of progress in the field: BioCreative (Blaschke *et al*., 2004) and Coling BioNLP (Collier *et al*., 2004).

In this paper we present a maximum entropy-based system for NER in biomedical abstracts which was entered in both of the above evaluations. Our system was originally designed for the BioCreative evaluation and was then adapted for the BioNLP task. We describe our system in detail, including its exhaustive use of local context as well as exploitation of a variety of external

Copyright © 2005 John Wiley & Sons, Ltd.

resources, including parsing, Google web-querying and gazetteers. We present our results in both evaluations and consider how the quality of the data affected the results. We found that performance in the tasks was more reflective of data quality than task difficulty. We discuss ways of improving annotation to provide maximal performance for machine learning systems.

### The tasks

The BioCreative NER task required participants to identify a single entity 'NEWGENE' in biomedical abstracts. This entity corresponded roughly to gene and protein names. Organizers provided 10 000 sentences from MEDLINE abstracts as training data and 5000 sentences as evaluation data. The average number of entities per sentence was roughly similar in both training and evaluation data (approximately 1.19).

The BioNLP NER task required participants to identify the five NEs 'protein', 'DNA', 'RNA', 'cell line' and 'cell type' in medical abstracts. The task was based on the GENIA corpus (Ohta *et al.*, 2002), a corpus of MEDLINE abstracts annotated for approximately 35 NE classes involved in biological reactions relating to transcription factors in human blood cells. The original set of NEs was collapsed into the above five by merging specific classes, such as 'protein molecule', 'protein family or group' and 'protein substructure', into broader classes ('protein') and dropping other classes such as 'body part' and 'virus' completely; the nested annotations contained in the original corpus were also removed for simplicity. The organizers did not say whether the adaptation of the corpus for the BioNLP task was done automatically. The entire GENIA corpus of 18 546 sentences was provided as training data, and an additional 3856 sentences as evaluation data. The average number of NEs per sentence was quite different between the training and evaluation data (for 'protein' 1.63 in training vs. 1.34 in testing; for 'DNA' 0.51 vs. 0.27; for 'RNA' 0.05 vs. 0.03; for 'cell line' 0.20 vs. 0.12; for 'cell type' 0.36 vs. 0.49).

Both BioNLP and BioCreative used the same exact-match scoring criterion, in which participants were penalized twice, both as a false positive (FP) and as a false negative (FN), for an answer with incorrect boundaries. For example, if the correct entity was *human interleukin-2 gene* and the system returned only *interleukin-2*, the former would be counted as a FN and the latter as a FP.

### System description

Our system is a maximum entropy Markov model (McCallum *et al.*, 2000) with a limited memory quasi-Newton maximizer based on a system used for the CoNLL 2003 shared task (Klein *et al.*, 2003). The system essentially uses a logistic regression model to classify each word, overlaid with a Viterbi-style algorithm to find the best sequence of classifications. Maximum entropy models have been used with much success in NER tasks and are known for their ability to incorporate a large number of overlapping features. For both evaluations we devoted most of our efforts to finding useful features for the NEs required. The final system makes exhaustive use of clues within the sentence including character substrings, words, word shapes, and detection of abbreviations, as well as using longer-distance information obtained from the surrounding abstract and relations obtained by parsing, and various external resources, including a Google web-querying technique, the TnT part-of-speech tagger (Brants, 2000) and a gazetteer. We normalized names of months and days of the week to lower case, and mapped the British spellings of a few common medical terms to their American equivalents. In the following sections we describe our full feature set.

We outline first the features utilizing the local context, and second the features corresponding to external resources and larger context. We also describe a postprocessing phase aimed at reducing boundary errors. Our final systems for both evaluations employed over 1.25 million features.

### Local features

We used a variety of features describing the immediate context of each word, including the word itself, the previous and next words, bi-grams of the current word and next word and the current word and previous word, character $n$-grams up to a length of six, word shapes, and features describing the named entity tags assigned to the previous words. 'Word shapes' refer to mappings of words

Copyright © 2005 John Wiley & Sons, Ltd.

to simplified representations that encode attributes such as length, and whether the word contains capitalization, numerals, Greek letters, and so on. We also incorporated part-of-speech (POS) tags from the TnT tagger trained on the GENIA gold standard for POS in biomedical text. We made use of abbreviation matching to ensure consistency of labels between an abbreviation and its long form. A list of abbreviations and long forms was extracted from the data using the method of Schwartz and Hearst (2003), then all occurrences of the short and long forms in the data were labeled as such (for BioNLP, we combined the list with the short and long forms from the BioCreative data). Features referencing these labels were then included in the classifier. Following (Kazama *et al*., 2002) we added disjunctive word features. Lastly, a parentheses-matching feature that signalled when one parenthesis was classified differently from its pair was added, in an

**Table 1.** Local features

| | |
|---|---|
| Word features | $w_i$, $w_{i-1}$, $w_{i+1}$ |
| | Last 'real' word (BioCreat. only) |
| | Next 'real' word (BioCreat. only) |
| | Disj. of four prev words (BioNLP-5) |
| | Disj. of four next words (BioNLP-5) |
| Bigrams | $w_i + w_{i-1}$ |
| | $w_i + w_{i+1}$ |
| TnT POS | $POS_i$, $POS_{i-1}$, $POS_{i+1}$ |
| Character substrings | Up to a length of six (BioNLP - prefix/suffix only) |
| Abbreviations | $abbr_i$ |
| | $abbr_{i-1} + abbr_i$ |
| | $abbr_i + abbr_{i+1}$ |
| | $abbr_{i-1} + abbr_i + abbr_{i+1}$ |
| Word shape | $shape_i$, $shape_{i-1}$, $shape_{i+1}$ |
| | $shape_{i-1} + shape_i$ |
| | $shape_i + shape_{i+1}$ |
| | $shape_{i-1} + shape_i + shape_{i+1}$ |
| TnT POS + word | $w_i + POS_i$ |
| | $w_{i-1} + POS_i$ |
| | $w_{i+1} + POS_i$ |
| Word shape + word | $w_{i-1} + shape_i$ |
| | $w_{i+1} + shape_i$ |
| Shape + word disj (BioNLP only) | $shape_i$ + disj of 5 previous words |
| | $shape_i$ + disj of 5 next words |
| Previous NE | $NE_{i-1}$ |
| | $NE_{i-2} + NE_{i-1}$ |
| | $NE_{i-3} + NE_{i-2} + NE_{i-1}$ (BioNLP only) |
| | $NE_{i-4} + NE_{i-3} + NE_{i-2} + NE_{i-1}$ (BioNLP only) |
| Previous NE + word | $NE_{i-1} + w_i$ |
| Previous NE + POS | $NE_{i-1} + POS_{i-1} + POS_i$ |
| | $NE_{i-2} + NE_{i-1} + POS_{i-2} + POS_{i-1} + POS_i$ |
| | $NE_{i-3} + NE_{i-2} + NE_{i-1} + POS_{i-3} + POS_{i-2} + POS_{i-1} + POS_i$ (BioNLP only) |
| Previous NE + abbr | $NE_{i-1} + abbr_{i-1} + abbr_i$ |
| | $NE_{i-2} + NE_{i-1} + abbr_{i-2} + abbr_{i-1} + abbr_i$ |
| Previous NE + shape | $NE_{i-1} + shape_i$ |
| | $NE_{i-1} + shape_{i+1}$ |
| | $NE_{i-1} + shape_{i-1} + shape_i$ |
| | $NE_{i-2} + NE_{i-1} + shape_{i-2} + shape_{i-1} + shape_i$ |
| PrevNE + shape + POS (BioNLP only) | $NE_{i-2} + NE_{i-1} + POS_{i-2} + POS_{i-1} + POS_i + shape_i$ |
| | $NE_{i-3} + NE_{i-2} + NE_{i-1} + POS_{i-3} + POS_{i-2} + POS_{i-1} + POS_i + shape_i$ |
| Paren-matching | Signals when one bracket in a pair has been assigned a different tag than the other in a window of four words |

Copyright © 2005 John Wiley & Sons, Ltd. 

effort to eliminate errors where the tagger classified matching parentheses differently. We combined all of the above base-level features in various ways. The full set of local features is outlined in Table 1.

### External resources and larger context

The features described here comprise various external resources, including gazetteers, a web-querying technique and relations obtained by parsing. The basic assumption behind, and motivation for, using external resources is that there are instances in the data where contextual clues do not provide sufficient evidence for confident classification. In such cases, external resources may bridge the gap, either in the form of word lists known to refer to genes (gazetteers) or through examination of other contexts in which the same token appears and the exploitation of more indicative contexts (as with web-querying and use of surrounding text, such as abstracts).

### Deep syntax features

Our system benefits from relational information obtained by parsing. While it has been stated that full parsing of biomedical text is beyond current technology, we were able to successfully parse the BioNLP training and evaluation corpora using the Stanford Parser (Klein and Manning, 2003) operating on the TnT POS tags. Since we did not have parsed biomedical text with which to train the parser, we used parsed *Wall Street Journal*; we believe that the unlexicalized nature of the Stanford parser made it suitable for parsing data from a different domain. For each word that appeared in a noun phrase, the head and governor of the noun phrase were extracted. These features were not useful in BioCreative because it involved identification of only one entity, but they were useful for BioNLP, where one had to disambiguate between similar classes; Shen *et al.* (2003) and Nobata *et al.* (1999) also benefit from use of head noun features with the GENIA entities. This disambiguation requires longer distance information and a better representation of the context in which the word appears. For instance, the word *phosphorylation* occurs in the training corpus 492 times, in 482 of which it was classified as 'other'. However, it was the governor of 738 words, of which 443 were 'protein', 292 were 'other' and only three were 'cell line'.

### Abstract

A number of NER systems have made effective use of how the same token was tagged in different parts of the same document (Mikheev *et al.*, 1999; Curran and Clark, 2003). A token which appears in an unindicative context in one sentence may appear in a very obvious context in another sentence in the same abstract. To leverage this, we tagged each abstract twice, providing for each token a feature indicating whether it was tagged as an entity elsewhere in the abstract. For BioCreative we were provided only single sentences from abstracts; we used cgi scripts to automatically obtain the corresponding full abstracts from MEDLINE. In a practical application this would be unnecessary, since one would always have the full abstract. Abstract information was only useful when combined with information on frequency.

### Web

As the largest corpus in existence, the web has been used effectively in a variety of NLP tasks (Keller and Lapata, 2003; Grefenstette, 1999; Markert *et al.*, 2003). In our use of the web we built several contexts indicative of target entities, such as 'X gene' or 'X antagonist' for genes, 'X mRNA' for RNA, or 'X ligation' for proteins. We then substituted the variable 'X' with potential entities and submitted the resulting patterns to the web. We used the number of hits obtained for each pattern to build a feature for the classifier. While the underlying principle was the same, the indicative contexts as well as the input X to such patterns differed in the two tasks. In both cases we submitted the pattern instantiations to the web using the Google API.

For BioCreative, we built patterns for each entity X identified as a gene by an initial run of the tagger. If at least one of the patterns returned more than zero hits, the string was assigned a 'web' value for the web feature. The classifier was then run again; this time incorporating the web feature. Using web-querying only on likely candidates for genes as identified by an initial

Copyright © 2005 John Wiley & Sons, Ltd.

run of the tagger was more efficient than using it on all words. However, this method does not contribute to improving recall. In the BioNLP task, we experimented with a different approach. We built indicative contexts for each of the five classes to be recognised and for each word X that had a frequency lower than 10, as estimated from the British National Corpus (BNC), a 100 million-word corpus taken from a wide variety of sources (Kilgarriff, 1997); we submitted the instantiation of each pattern to the web. The pattern that returned the highest number of hits determined the feature value (e.g. 'web-protein', or 'web-RNA'). If no hits were returned by any pattern, a value 'O-web' was assigned. The same value was assigned to all words whose frequency was higher than 10; using another value for words with high frequency did not improve performance. This method proved less successful than the one used in our BioCreative system; it is unclear whether this is due to the method or to differences in the BioNLP task. In future work we will reproduce the same experiments on the two datasets in order to answer this question.

### Gazetteer

Our gazetteer was compiled from lists of gene names from biomedical sites on the Web (such as Locus Link) as well as from the Gene Ontology and the data provided for BioCreative Tasks 1A and 1B. The gazetteer was cleaned by removing single character entries ('A', '1'), entries containing only digits or symbols and digits ('37', '3-1'), and entries containing only words that could be found in the English dictionary CELEX ('abnormal', 'brain tumour'). The final gazetteer contained 1 731 581 entries.

### Frequency

We sought to incorporate information on frequency primarily as a way to weight information from external resources and to a lesser extent to indicate independently which tokens might be names. Because more frequent words are more likely to be ambiguous and less frequent words are far less likely to be ambiguous, we assumed that information from external resources would be of greater use for low-frequency words. We therefore assigned to each word a frequency category corresponding to the number of times the word was seen in a corpus. For BioCreative the corpus used was the BioCreative training data. For BioNLP, we improved on this by using counts from the BNC. We found that the frequencies obtained from the BNC were more intuitive than frequencies from a medical corpus.

### Postprocessing

For BioCreative, we found that many of our errors stemmed from gene boundaries and addressed this issue in several ways. We removed genes containing mismatched parentheses from our results. We also found that we obtained different boundaries when we ran the classifier forwards vs. backwards (reversing the order of the words) and obtained a significant improvement by simply combining the two sets of results and then keeping only the shorter entity in cases where one entity was a substring of another. We found that this postprocessing was highly valuable and added approximately 1% to our F-score. For BioNLP, we found that postprocessing was not useful because running the classifier forwards produced poor results and because mismatched parentheses were less of a problem.

### Results and analysis

The performance of the system in both tasks is shown in Tables 2 and 3; the system gets an overall F-score of 83.2 for the BioCreative NER task and

**Table 2.** Results for BioCreative

| | Precision | Recall | F-Score |
|---|---|---|---|
| Gene/protein | 82.8 | 83.5 | 83.2 |

**Table 3.** Results for BioNLP

| | Precision | Recall | F-Score |
|---|---|---|---|
| Protein | 77.4 | 68.5 | 72.7 |
| DNA | 66.2 | 69.6 | 67.9 |
| RNA | 72.0 | 65.9 | 68.8 |
| Cell line | 59.0 | 47.1 | 52.4 |
| Cell type | 62.6 | 77.0 | 69.1 |
| Overall | 71.62 | 68.6 | 70.1 |

Copyright © 2005 John Wiley & Sons, Ltd.

70.1 for the BioNLP task. Our system compared well with other systems in both evaluations. Comparison to other results published on GENIA NE subsets is difficult, because groups choose different subsets of GENIA entities and often evaluate on private corpora. Shen *et al.* (2003) report an F-score of 66 on a 24-NE task using version 3 of GENIA to evaluate. Collier *et al.* (2000) and Koichi and Collier (2003) attempt a 10-NE task, using a private corpus to evaluate, and report F-scores of 74 and 73. We have analysed our sources of error for both BioCreative and BioNLP in depth in Dingare *et al.* (2004) and Finkel *et al.* (2004); these include a large percentage of boundary errors (over 30% for both tasks), a smaller number of errors due to coordination, and some errors due to acronyms and tokens, whose orthographic form might suggest they were entities but were in fact measures or belonged to other entity categories; also a number of errors due to low-frequency words or words not encountered in the training data. However, we would like to focus here on the quality of training and evaluation data as a key factor leading to low performance. The 13-point discrepancy between performance in BioCreative and BioNLP might be partially explained by the varying task difficulty: BioNLP requires recognition of five entities, while BioCreative requires only one; BioNLP also requires disambiguation of systematically ambiguous gene and protein names. However, task difficulty does not appear to be the primary factor leading to lower performance. To demonstrate this, we evaluated the system's performance on the BioNLP data for the task of identifying a single class. When we eliminated the 'cell line' and 'cell type' categories and combined the 'DNA', 'RNA' and 'protein' categories into a single class, we obtained an F-score of 74.4. This figure is substantially below the performance of 83.2 obtained for the roughly equivalent 'NEWGENE' class in BioCreative. Rather than task difficulty, lower performance in BioNLP stems from higher inconsistency in the annotation of the BioNLP data. In saying this, we refer not only to errors in the evaluation data, which resulted in lower scores, but equally to inconsistencies in the training data, which caused the system to learn incorrect patterns. Two of the authors independently reviewing 50 of the system's errors found that 34–35 of these could be attributed to inconsistent annotation of the training or evaluation data. We are not biologists; we based our judgements of inconsistency on similarity of context. However, the example pairs we list below are so similar that we do not think the annotation inconsistencies are due to biological subtleties.

### Data annotation

Approximately one-third of the system's errors were due to highly variable annotation of frequent terms such as *lymphocyte, T cell* and *B cell*; these were variously annotated as 'cell type' and as 'O' (i.e. not in an entity). In example (1) below from the evaluation data, our system labelled *lymphocytes* as a 'cell type' and was penalized for a FP. However, our annotation is consistent with example (2) which appeared only two sentences later in the evaluation data; *lymphocytes* is annotated as a 'cell type' there.

(1) ... content of cAMP was also decreased in lymphocytes by 33%.
(2) ... simultaneous alteration in the cAMP content was observed in *lymphocytes*.

Parallel problems occurred with the frequent terms *hormone* and *receptor*, which were variously annotated as 'protein' and 'O'. In example (3) from the evaluation data, our system labelled *receptors* as 'O' rather than 'protein' and was penalized for a FN; however, our annotation mirrors example (4), which appeared in the training data.

(3) Concentration of the *receptors* to 1.25 (OH) 2D3 was elevated up to 39.7 fmole/mg after 1week ...
(4) Concentration of receptors of hormonal form of 1,25 (OH) 2D3 was found to be minimal ...

In a smaller proportion of cases, entities were variably annotated either 'DNA' or 'protein'. In example (5) below, which appeared in the evaluation data, *kappa B enhancer* was labelled as 'protein', while in example (6), which appeared in the training data, it was labelled as 'DNA'. Variation in labelling between 'DNA' and 'protein' also occurred with *enhancer elements*.

(5) These kappa B-specific proteins ... interact with the functional *kappa B enhancer* present in the IL-2R alpha promoter.
(6) ... nuclear NF-kappa B is necessary to activate the *kappa B enhancer* ...

Inconsistent annotation of premodifiers also caused a small number of errors. In examples (7),

Copyright © 2005 John Wiley & Sons, Ltd.

(9) and (11), which appeared in the evaluation data, the modifiers *human, inducible* and *unrearranged* were included in the entities 'DNA', 'protein' and 'DNA', respectively, while in the parallel examples (8), (10) and (12), which appeared in the training data, they were excluded. Our system left out the modifiers, as in the training data, and was penalized for both FPs and FNs.

(7) Kappa B-specific DNA binding proteins: role in the regulation of *human interleukin-2 gene* expression.
(8) Instead, signal transduction to the human *IL-2 gene* became disrupted.
(9) Mutation of a kappa B core sequence ... blocks the specific binding of two *inducible cellular factors*.
(10) [Sequence analysis revealed] several putative binding sequences for inducible *transcription factors* ...
(11) Different fragments of *unrearranged human variable region* ... were used for ... *in vitro* transcription ...
(12) ... hGATA-3 may be involved in the regulation of the unrearranged *TcR delta gene* expression ...

Some cases of inconsistent annotation were due to cancer terms, such as *neoplasm, tumor* and *carcinoma*, which were annotated either as 'cell type' or 'O'; we assume that this is because these terms are ambiguous between cell types and disease names.

(13) ... the authors studied specimens of *breast carcinomas* from 60 consecutive female patients.
(14) Inflammatory infiltrates were analysed in tissue sections of 76 breast carcinomas ...

There was also uncertainty as to whether gene systems, core sequences, and stretches of DNA described by numerical location (e.g. −206 to −195) should count as 'DNA' entities. Finally, there was highly variable annotation of coordination.

Overall, the quality of data in the BioCreative evaluation appeared to be significantly higher and did not feature the systematic inconsistencies of the BioNLP data (keeping in mind that the BioCreative annotation task was also significantly easier). BioCreative's innovation of enumerating several alternative correct boundaries reduced spurious boundary errors. However, there were some inconsistencies in the BioCreative data as well. In a few cases organism names appearing in prepositional phrases after gene names were annotated as if they were premodifiers [as in (15)], while in other cases they were not [as in (16)].

(15) Transcriptional regulation of *SUP35* and *SUP45 in Saccharomyces cerevisiae.*
(16) Expression of the ... protein Bax under the control of a *GAL10* promoter in Saccharomyces cerevisiae resulted in ...

The annotation of *mutations* was also inconsistent; the participants were given instructions not to annotate *mutations* as genes and were given the example *p53 mutations*; but in the training data there were 25 instances of *mutations* annotated as genes, including *p53 mutations*.

### Improving biomedical annotation

That the task of biomedical NER is more difficult than NER in the traditional newswire domain (with its standard entities of 'PERSON', 'LOCATION' and 'ORGANIZATION') is obvious from the numbers; the highest score in the CoNLL 2003 NER task (Sang and De Meulder, 2003; which used the same scoring metric as BioNLP and BioCreative) was 88.8%, five points higher than the highest score in BioCreative and 18 points higher than our score in BioNLP. What must be noted is that the difficulty of the domain has an effect on both the annotation of the data and the performance of the system. In a difficult domain where language is convoluted and names are long and complex, data annotation is more difficult. This is demonstrated by results on interannotator agreement; while interannotator agreement for the MUC-7 NER task in the newswire domain was measured at 97% (Marsh and Perzanowski, 1998), the few studies of interannotator agreement in the biomedical domain have shown interannotator agreement to be substantially lower, with F-scores in the range of 0.87 (Hirschman, 2003) to 0.89 (Demetriou and Gaizauskas, 2003). In order to represent the state of progress in biomedical NER accurately, evaluations must focus as much on improving biomedical data annotation as on improving systems. We note that while the use of annotation guidelines has become standard practice, particularly for complex annotation tasks, the annotation of the BioCreative data did not use annotation guidelines. We also know of no guidelines used in the annotation of the GENIA

Copyright © 2005 John Wiley & Sons, Ltd. 

data used in the BioNLP task. The adoption of annotation guidelines in a domain notorious for its complexity, and where interannotator agreement is known to be low, seems to be a promising direction for improvement.

Annotation guidelines must address the proper annotation of premodifiers, constructing rules to distinguish the premodifiers that are necessary to annotate. They must also specify how to annotate coordinated entities, distinguishing between the varieties of coordinations. Next, they must establish whether to annotate high-level categories. It may be that the variability in the annotation of words like *receptor* and *hormone* was due to the fact that receptors and hormones are types of protein containing thousands of instances. Finally, annotation guidelines must decide ambiguous cases of class membership, such as whether DNA sequences are examples of 'DNA' entities and whether tumours are 'cell types'.

### Conclusions

We have presented a machine learning system for biomedical NER and presented its performance in the two biomedical NER evaluations to date. Our system's rich feature set, including exhaustive use of local features and a variety of external resources, leads to state-of-the-art performance. Our system also adapts rapidly to new NE sets, as illustrated by our adaptation to the BioNLP task.

Unfortunately, state-of-the-art performance in biomedical NER continues to lag behind the high-80s figures that the field has come to expect. The BioNLP organizers may have had this gap in mind when they emphasized that participants should focus on deep knowledge sources, such as co-reference resolution and use of dependency relations over 'widely used lexical-level features (POS, lemma, orthographic, etc.)'. However, both BioNLP and BioCreative showed that external resources led to improvements of only 1–2%. Our error analysis showed that consistent annotation might have led to a 70% reduction in error rate. While the proper exploitation of external resources and deep processing remains an avenue to be explored, we believe it cannot compare to the gains that might result from consistently annotated data. The challenge for future evaluations is to use and publish annotation guidelines, to measure and report figures for interannotator agreement, and to pursue improvements in annotation of biomedical data alongside improvements in systems.

### Acknowledgements

This work was supported by a Scottish Enterprise Edinburgh–Stanford Link Grant (R36759) as part of the SEER project, and by the National Science Foundation under the Knowledge Discovery and Dissemination program.

### References

Blaschke C, Hirschman L, Yeh A (eds). 2004. *Proceedings of the BioCreative Workshop, Granada*; **http://www.pdg.cnb.uam.es/BioLINK/workshop_BioCreative_04/handout/**

Brants T. 2000. TnT — a statistical part-of-speech tagger. In *Proceedings of the Sixth Applied Natural Language Processing Conference ANLP-2000*, Seattle, WA **6**: 224–231.

Collier N, Kim J, Tateisi Y, Ohta T, Tsuruoka Y (eds). 2004. *Proceedings of the International Joint Workshop on Natural Language Processing in Biomedicine and Its Applications, Geneva, Switzerland*. **http://www.genesis.ch/~natlang/JNLPBAO4**.

Collier N, Nobata C, Tsujii J. 2000. Extracting the names of genes and gene products with a hidden Markov model. In *Proceedings of the 18th International Conference on Computational Linguistics (Coling 2000)*, Saarbruecken, Germany; pp. 201–207.

Curran JR, Clark S. 2003. Language independent NER using a maximum entropy tagger. In *Proceedings of the 7th Conference on Natural Language Learning (CoNLL-03), Edmonton, Canada*; 164–167.

Demetriou G, Gaizauskas R. 2003. Corpus resources for development and evaluation of a biological text mining system. In *Proceedings of the Third Meeting of the Special Interest Group on Text Mining, Brisbane, Australia*; **http://www.pdg.cnb.uam.es/BioLink/SpecialInterestText Mining/PRESENTATIONS/rob_g.ppt**

Dingare S, Finkel J, Nissim M, Manning C, Alex B. 2004. Exploring the boundaries: Gene and protein identification in biomedical text. In *Proceedings of the BioCreative Workshop, Granada*; **http://www.pdg.cnb.uam.es/BioLINK/workshop_BioCreative_04/handout/**

Finkel J, Dingare S, Nguyen H, Nissim M, Manning C. 2004. From syntax to the web. In *Proceedings of the International Joint Workshop on Natural Language Processing in Biomedicine and Its Applications*, at CoLing 2004, Geneva, Switzerland; pp. 88–91.

Fukuda K. 1998. Toward information extraction: Identifying protein names from biological papers. In *Proceedings of the Pacific Symposium on Biocomputing* 705–716.

Grefenstette G. 1999. TheWWW as a resource for example-based MT tasks. In *Proceedings of ASLIB'99, Translating and the Computer* 21, London.

Hirschman L. 2003. Using biological resources to bootstrap text mining. Presentation to the Massachusetts Biotechnology Council Informatics Committee; **http://www.e-biosci.org/sept/Hirschman.pdf**

Copyright © 2005 John Wiley & Sons, Ltd. 

Kazama J, Makino T, Ohta Y, Tsujii J. 2002. Biomedical name recognition: Tuning support vector machines for biomedical named entity recognition. In *Proceedings of the ACL 2002 Workshop on Natural Language Processing in the Biomedical Domain* 1–8.

Keller F, Lapata M. 2003. Using the web to obtain frequencies for unseen bigrams. *Comput Linguist* **29**(3): 459–484.

Kilgarriff A. 1997. Putting frequencies in the dictionary. *Int J Lexicogr* **10**(2): 135–155.

Klein D, Manning C. 2003. Accurate unlexicalized parsing. *Proceedings of the 41st Annual Meeting of the Association for Computational Linguistics (ACL, 2003)*, Sapporo, Japan, pp. 423–430.

Klein D, Smarr J, Nguyen H, Manning CD. 2003. Named entity recognition with character-level models. *Proceedings of the 7th Conference on Natural Language Learning (CoNLL 2003)*, Edmonton, Canada; pp. 180–183.

Koichi T, Collier N. 2003. Bio-medical entity extraction using support vector machines. In *Proceedings of the Workshop on Natural Language Processing in Biomedicine, held as part of the 41st Annual Meeting of the Association for Computational Linguistics (ACL, 2003)*, Sappoco, Japan, July 7–12, 2003.

Markert K, Nissim M, Modjeska N. 2003. Using the web for nominal anaphora resolution. In *Proceedings of the Workshop on the Computational Treatment of Anaphora held as part of the 11th Conference of the European Chapter of the Association for Computational Linguistics (EACL, 2003)*, Budapest, Hungary, April 12–17, 2003. Dale R, van Deemter K, Mitkov R (eds) 39–46.

Marsh E, Perzanowski D. 1998. MUC-7 evaluation of IE technology: Overview of results. In *Message Understanding Conference Proceedings* **7;http://www.itl.nist.gov/iaui/894.02/related_projects/muc/proceedings/muc_7_proceedings/marsh_slides.pdf**

McCallum A, Freitag D, Pereira F. 2000. Maximum entropy markov models for information extraction and segmentation. In *Proceedings of the 17th International Conference on Machine Learning*.

Mikheev A, Moens M, Grover C. 1999. Named entity recognition without gazetteers. In *Proceedings of the 9th Conference of the European Chapter of the Association for Computational Linguistics (EACL '99)*, Bergen, Norway, June 8–12, 1999, pp. 1–8.

Nobata C, Collier N, Tsujii J. 1999. Automatic term identification and classification in biology texts. In *Proceedings of the 5th Natural Language Processing Pacific Rim Symposium (NLPRS '99)*, Beijing, China, November 5–7, 1999; pp. 369–374.

Ohta T, Tateisi Y, Mima H, Tsujii J. 2002. GENIA corpus: an annotated research abstract corpus in molecular biology domain. In *Proceedings of the Human Language Technology Conference*, San Diego, CA, USA, March 24–27, 2002.

Sang EFTK, De Meulder F. 2003. Introduction to the CoNLL-2003 shared task: language-independent named entity recognition. In *Proceedings of CoNLL-2003* 142–147.

Schwartz A, Hearst M. 2003. A simple algorithm for identifying abbreviation definitions in biomedical text. In *Pacific Symposium on Biocomputing, Kauai*.

Shen D, Zhang J, Su GZJ, CTan. 2003. Effective adaptation of hidden Markov model-based named entity recognizer for biomedical domain. In *Proceedings of the Workshop on Natural Language Processing in Biomedicine, held as part of the 41st Annual Meeting of the Association for Computational Linguistics (ACL, 2003)*, Sapporo, Japan, July 7–12, 2003.

Copyright © 2005 John Wiley & Sons, Ltd. 
